# Supplementary material for: Reduction in medical costs for cardiovascular diseases through innovative health awareness projects in a rural area in Japan
Source: PLoS One. 2022 Nov 16;17(11):e0277600. doi: 10.1371/journal.pone.0277600 (PMC9668126; doi:10.1371/journal.pone.0277600)
Supplement: S1 Appendix — (DOCX) [file pone.0277600.s001.docx]

**S1 Appendix**

**Risk score to estimate the 10-year incidence risk of CHDs**

The detail of this method has been provided by Kinoshita M~~,~~ et al. [12]. The risk score model includes the following variables as risk factors: age, sex, smoking status, HDL-C, LDL-C, blood pressure, impaired glucose tolerance, and a family history of early onset coronary heart disease. Table S1 lists all risk factors and points scored for weighing risks.

**Table S1. Points assigned to CHD risk factor categories.**

| Risk factor | Category | Points |
| --- | --- | --- |
| Age (years) | 35 to <45 | +30 |
|  | 45 to <55 | +38 |
|  | 55 to <65 | +45 |
|  | 65 to <70 | +51 |
|  | ≥70 | +53 |
| Sex | Female | −7 |
| Current smoking | Yes | +5 |
| HDL-C (mg/dL) | 40 to <60 | −5 |
|  | ≥60 | −6 |
| LDL-C (mg/dL) | 100 to <140 | +5 |
|  | 140 to <160 | +7 |
|  | 160 to <180 | +10 |
|  | ≥180 | +11 |
| Blood pressure (SBP/DBP) (mmHg) | <120 and <80 | −7 |
|  | 140 to <160 and/or 90 to <100 | +4 |
|  | ≥160 and/or ≥100 | +6 |
| Impaired glucose tolerance | Yes | +5 |
| Family history of early onset CHDs | Yes | +5 |

**Table S2. Predicted 10-year risk of CHD by risk score**

| Total score | Predicted risk |
| --- | --- |
| <36 | 0.005 |
| 36 to <41 | 0.01 |
| 41 to <46 | 0.02 |
| 46 to <51 | 0.03 |
| 51 to <56 | 0.05 |
| 56 to <61 | 0.09 |
| 61 to <66 | 0.14 |
| 66 to <71 | 0.22 |
| ≥71 | 0.281† |

* Kinoshita M et al. has reported <0.01 with a median value of 0.005 [12].

†Kinoshita M et al. has reported ≥0.28 with a minimum value of 0.281 [12].

**Risk score to estimate the 10-year incidence risk of stroke**

The estimation method has been presented in detail by Yatsuya H~~,~~ et al. [13]. The included risk factors in the risk score model are as follows: age, sex, smoking status, blood pressure, obesity, and diabetes mellitus. Table S3 shows all risk factors along with points used to weigh risks.

**Table S3. Points assigned to stroke risk factor categories~~.~~**

| Risk factor | Category | Points |
| --- | --- | --- |
| Age (years) | 45 to < 49 | +5 |
|  | 50 to < 54 | +6 |
|  | 55 to < 59 | +12 |
|  | 60 to < 64 | +16 |
|  | ≥ 65 | +19 |
| Sex | Male | +6 |
| Current smoking | Male | +4 |
|  | Female | +8 |
| Body mass index (kg/m^2^) | 25 to <30 | +2 |
|  | ≥30 | +3 |
| Blood pressure (SBP/DBP) (mmHg) |  |  |
| No antihypertension medication | 120 to <130 / 80 to <85 | +3 |
|  | 130 to <140 / 85 to <90 | +6 |
|  | 140 to <160 / 90 to <100 | +8 |
|  | 160 to <180 / 100 to <110 | +11 |
|  | ≥ 180 / ≥110 | +13 |
| On antihypertensive medication | <120 / < 80 | +10 |
|  | 120 to <130 / 80 to < 85 | +10 |
|  | 130 to <140 / 85 to < 90 | +10 |
|  | 140 to <160 / 90 to <100 | +11 |
|  | 160 to <180 / 100 to <110 | +11 |
|  | ≥ 180 / ≥110 | +15 |
| Diabetes mellitus | Yes | +7 |

**Table S4. Predicted 10-year risk of stroke by risk score**

| Total score | Predicted risk* |
| --- | --- |
| <10 | 0.005 |
| 11 to <18 | 0.015 |
| 18 to <23 | 0.025 |
| 23 to <26 | 0.035 |
| 26 to <28 | 0.045 |
| 28 to <30 | 0.055 |
| 30 | 0.065 |
| 31 to <33 | 0.075 |
| 33 | 0.085 |
| 34 | 0.095 |
| 35 to <37 | 0.110 |
| 37 to <40 | 0.135 |
| 40 to <43 | 0.175 |
| ≥43 | 0.250 |

* Yatsuya H et al. has provided the ranges of risk; we used the median value of the range. We also used 0.005 for minimum risk (at <0.01) and 0.250 for maximum risk (at ≥0.200) [13].
