# Supplementary material for: Reduction in medical costs for cardiovascular diseases through innovative health awareness projects in a rural area in Japan
Source: PLoS One. 2022 Nov 16;17(11):e0277600. doi: 10.1371/journal.pone.0277600 (PMC9668126; doi:10.1371/journal.pone.0277600)
Supplement: S2 Appendix — (DOCX) [file pone.0277600.s002.docx]

**S2 Appendix**

**Factors related to changes in risk scores after the removal of cases with missing records**

We removed participants with missing records of risk factors (the number of missing values of each factor is shown in Table 1) and examined the impact of our assumption that participants possess no risk in instances where records are missing for risk factors. All resultant estimates were similar, regardless of whether such patients were included or excluded in calculations (Table 2A and S5A and Table 2B and S5B).

**Table S5. Factors related to risk scores**

**(A) CHDs**

|  | **Without CHAP** | | | | **With CHAP** | | | |
| --- | --- | --- | --- | --- | --- | --- | --- | --- |
|  | Est. | S.E. | t | p | Est. | S.E. | t | p |
| **(Intercept)** | 0.220 | 0.763 | 0.289 | 0.773 | 0.203 | 0.560 | 0.362 | 0.717 |
| **Elapsed years** | 2.645 | 0.324 | 8.160 | <0.001 | 0.409 | 0.077 | 5.337 | <0.001 |
| **Sex** | −2.017 | 0.300 | 6.729 | <0.001 | −2.551 | 0.232 | 11.005 | <0.001 |
| **Age at the start of each period (year)** | 0.221 | 0.019 | 11.665 | <0.001 | 0.252 | 0.014 | 18.031 | <0.001 |
| **Temperature on the checkup date** | 0.104 | 0.064 | 1.628 | 0.104 | −0.260 | 0.043 | 6.075 | <0.001 |
| **CHAP Preparation (degree)** | −3.979 | 0.555 | 7.163 | <0.001 |  |  |  |  |
| **Risk score of previous year** | 0.766 | 0.015 | 51.776 | <0.001 | 0.738 | 0.011 | 68.770 | <0.001 |

**(B) Stroke**

|  | **Without CHAP** | | | | **With CHAP** | | | |
| --- | --- | --- | --- | --- | --- | --- | --- | --- |
|  | Est. | S.E. | t | P | Est. | S.E. | t | p |
| **(Intercept)** | −1.477 | 0.683 | 2.162 | 0.031 | −0.685 | 0.460 | 1.488 | 0.137 |
| **Elapsed years** | 1.157 | 0.290 | 3.992 | <0.001 | 0.171 | 0.064 | 2.671 | 0.008 |
| **Sex** | −2.153 | 0.276 | 7.799 | <0.001 | −2.392 | 0.203 | 11.804 | <0.001 |
| **Age at the start of each period (year)** | 0.204 | 0.015 | 13.193 | <0.001 | 0.194 | 0.010 | 18.974 | <0.001 |
| **Temperature on the checkup date (degree)** | 0.053 | 0.057 | 0.919 | 0.358 | −0.073 | 0.036 | 2.052 | 0.040 |
| **CHAP preparation** | −1.960 | 0.494 | 3.965 | <0.001 |  |  |  |  |
| **Risk score of previous year** | 0.734 | 0.017 | 43.792 | <0.001 | 0.731 | 0.012 | 62.302 | <0.001 |
