# Supplementary material for: Reduction in medical costs for cardiovascular diseases through innovative health awareness projects in a rural area in Japan
Source: PLoS One. 2022 Nov 16;17(11):e0277600. doi: 10.1371/journal.pone.0277600 (PMC9668126; doi:10.1371/journal.pone.0277600)
Supplement: Attachment — Submitted filename: response_to_reviewers_economic_impact_20May2022.docx [file pone.0277600.s003.docx]

Dear Reviewers,

Thank you for your careful reviews and constructive comments. We have revised the manuscript according to your suggestions and would be grateful if you would review the updated version.

Sincerely,

Ayako Shoji

**Reviewer #1**

***Comments to the Authors***

*Overall, the manuscript is well written. However, there are several points that should be improved in terms of potential additional analyses and justifications of the proposed methodology..*

**Author Response:** Thank you for the valuable comments. We have updated according to your suggestions as described below.

***Comment 1.*** *It should be clearly stated what is the novelty of this study and what it adds to the existing literature.*

**Author Response:** Thank you for the indication. We have added descriptions that explain the novelty of our study and the difference of methods and results from previous studies in Discussion section as follows:

Discussion section (Pages 23-24, Lines 261-276)

The novelty of this study is that it suggested the impact of a personalized interview based on individual results on decreasing CVD risks as well as reducing medical costs. CHAP is characterized by an annual checkup with comprehensive health check items and a single interview to provide information on possible lifestyle changes to solve problems immediately after the checkup~~. The program intends to decrease implementation costs and burden on participants.~~ , ~~This~~ which is different from ~~in contrast to~~ general population health approaches that usually are intense during a specific period or consist of regular education programs [9]. A similar low-intense program for lifestyle modification to CHAP is already offered. The Japanese nationwide health guidance program for lifestyle modification consists of an initial interview based on the results of health checkups followed by monitoring of healthy behavior and continuous support for three or more by instructors. However, it is offered only for high-risk persons few months after the checkups and its longitudinal clinical effect is unlikely to be clear [10,11,19]. Results from the present study suggest that even one personalized effort to raise awareness and encouragement in the whole participants in a year can prevent a deterioration in the laboratory parameters and an occurrence of CVDs.

***Comment 2.*** *It is not clear what kind of data were used. It is stated that data were obtained from the Iwaki cohort, so presumably the analysis was based on patient’s level data, but this is not stated. It maybe that aggregate data were used, but again, this is not stated or justified. This is somewhat explained in the limitation section, but it should be explained clearly and in greater details in the methods section.*

**Author Response:** Thank you for the indication. We found that our explanation was insufficient as you indicated, and then have added descriptions in Materials and Method section as follows:

Materials and Method section (Page 5, Lines 87-91)

The Iwaki cohort data ~~is~~ contains individual-based 3000 health-related items ~~individual-based~~ collected from ~~including~~ both healthy and ill persons in the same manner every year before and after the initiation of CHAP, which allowed us to compare the trends in health parameters between ~~patients~~ participants with and without CHAP and determine its effect (Fig 1B).

Materials and Method section (Page 6, Lines 109-110)

We calculated risk scores of CHDs and stroke that predict ~~We estimated~~ the 10-year incidence risks of ~~CHDs and stroke~~ those diseases.

Materials and Method section (Page 8, Lines 152-155)

Using generalized linear models and individual levels checkup data with calculated risk scores, we analyzed coefficients and evaluated factors associated with risk scores for each period (with and without CHAP), using the risk score as an explained variable.

Materials and Method section (Page 9, Lines 173-177)

Based on the individual predicted risk scores, we estimated the proportion of individuals per risk score and age group. We then multiplied the proportion by the number of residents per sex and age group ~~[14]~~ in Aomori [14] and incidence rates according to risk scores ~~to~~ for estimating~~e~~ the incidences of CHDs and stroke within 10 years after 2022.

***Comment 3.*** *The method section is not clear, a good way to explain the clinical and economic impact of the CHAP programme is a before and after econometric method. The authors should explain why this was not possible considering the data limitation.*

**Author Response:** Thank you for the important suggestion. We could not use a difference-in-difference method, because the cohort data that we used does not include persons who hadn’t participate CHAP after the initiation of CHAP. Alternatively, we used generalized linear models as considering risk factors of CVDs and differences among participants, but we did not compare between the periods before and after the initiation of CHAP by using one model, because we thought that CHAP is a program, which intend to change participants’ attitude and behavior and its effect too complex to consider in one model. We have added this explanation in Materials and Method, and Limitations section as follows:

Materials and Method section (Page 9, Lines 164-168)

We evaluated the trend of risk scores by using two models for periods before and after the initiation CHAP separately. Because CHAP was intended to change overall attitudes and behaviors of participants and it has been offered all participants since 2013, therefore we did not directly compare between the periods by using one model.

Limitations section (Page 26, Lines 311-313)

By using the individual-based exhaustive data, we also expect to directly compare between the periods before and after the initiation of CHAP as well as between participants of CHAP and non-participants.

***Comment 4.*** *The risk score to estimate the 10-year incidence risks of CHDs and stroke is referenced, but a brief explanation of what is it and how it works should be provided (perhaps as supporting information).*

**Author Response:** Thank you for the indication. We have added the explanation as a supporting information.

Materials and Method section (Page 6, Lines 113-116)

The equation of CHDs was obtained from the Japanese guideline for the prevention of atherosclerotic CHDs[12] and that of stroke was done from the JPHC study, a large-scale cohort study in Japan[13] (details were described in Supplementary Information) .

Materials and Method section (Page 7, Lines 131-132)

We assumed no risk and counted a score of 0 for every missing risk factor. Detail information is shown in Supporting information.

***Comment 5.*** *Costs are calculated by multiplying the incidence of CHDs and stroke by medical costs. The estimation is based on assumptions and not on a costing methodology. It is not clear why, if patents level data are available, costs were not estimated with, for instance a two-part model (accounting for 0 costs and skewed distribution of healthcare costs) or any other sound methodologies.*

**Author Response:** Thank you for the indication. As was answered your comment #2, we found that our explanation was unclear. In the cohort data that we used, there is no information to be used for estimating the amount of healthcare resource uses. We have added the description in Materials and Methods section and Limitation section as follows:

Materials and Method section (Page 5, Lines 92-94)

However, this cohort data does not contain the information of health resource utilization to be used for estimating health care costs.

Limitation section (Page 25, Lines 296-299)

We used medical costs per patient by referring to government statistics on national medical expenditure based on healthcare resource utilization for the management of stroke or CHDs, because the Iwaki cohort data does not include any data on healthcare resource utilization.

***Comment 6.*** *Table and figures could be presented in a more appealing way. E.g., in Table 1, numbers and percentages could be presented within one single cell. Similarly, mean and SD could be presented together. E.g., age in years in 2008 could be presented in the following way 56.97 (13.10). Figures 2 should be fully labelled to aid the reader.*

**Author Response:** Thank you for the significant comment. We have corrected Table 1 and Figure 2 as you indicated.

***Comment 7.*** *In the Conclusions, it is stated that the innovative project for awareness and encouragement contributes to a significant cost reduction in the treatment of CVDs and reduces the economic burden on the local government. In reality, this is based on assumptions as a formal econometric analysis was not conducted. It could be said, however, that the innovative project has the potential for cost reduction etc. etc.*

**Author Response:** Thank you for the important suggestion. We have changed the description as you proposed in Conclusion section as follows:

Conclusion section (Page 26, Lines 315-317)

We found that an innovative project for awareness and encouragement in the rural area has the potential for ~~contributes to a significant~~ cost reduction in the treatment of CVDs and then reduction in ~~reduces~~ the economic burden on the local government.

**Reviewer #2**

***Comments to the Authors***

*This study was revealed the effect of CHAP as an innovative project on the prevention of stroke and coronary heart disease as well as to contribute to a cost reduction in the treatment of these diseases. The concept of the present study was valuable but there are some concerns as described below.*

**Author Response:** Thank you for the evaluation and the important comments. We have updated according to your suggestions as described below.

***Comment 1.*** *In Table 1, the subjects appear to be younger after 2012 than before. Author described that “The mean age, which is an important risk factor of CHDs and stroke, did not show any significant change in either period but decreased by about 3 years around 2012” in page10, line 182-184.*

**Author Response:** Thank you for the important indication. We thought that your concern associated with your next comment #2 and then have added additional analyses according to your suggestion in comment #2. We are happy if you could see our response to comment #2.

***Comment 2.*** *In Table 1, the author showed significant trend changes in SBP, DBP, and HbA1C, before and after 2012, but I wonder if they are adjusted for age, because the mean age seems to be younger after 2012 than before.*

**Author Response:** Thank you for the significant suggestion. We additionally analyzed according to your suggestion and confirmed that the trends of SBP, DBP, and HbA1C during the period before and after 2012 did not significantly changed, even after the adjustment for age. We added the description about the results in Results section as follows:

Results section (Page 10, Lines 196-198)

Even after the adjustment for age, the trends in mean systolic and diastolic blood pressure and HbA1C levels remained significant as described above.

***Comment 3.*** *I don't understand how you could show “a more remarkable increase in risk factors (Table 2C).” in page16, line 207-208. Could you explain more about it?*

**Author Response:** Thank you for the indication. We have changed the description to clarify as follows:

Results section (Page 18, Lines 218-221)

A sensitivity analysis was conducted for the period from 2008 to 2012, defined as without CHAP, and confirmed that derived estimated coefficients were similar to base case analysis, ~~as well as a~~ which showed more remarkable increase in risk factors than sensitivity analysis (Table 2C).

***Comment 4.*** *In page 22; line 262-263, the author described that “the effect of CHAP was remarkable for SBP, DBP, and HbA1C, but not for other risk factors or lifestyle factors.”Is it possible that there was a bias in the content of lifestyle interview or guidance in CHAP? It would be better to mention standardization of instructional content.*

**Author Response:** Thank you for the significant suggestion. The process of CHAP has been published as a protocol that we referred in the present study. Lifestyle information is collected by using a same questionnaire every year and then we believe that there is only a limited bias arising from interviewers. We have explained it in Materials and Method section as follows:

Materials and Method section (Page 5, Lines 91-92)

Even participant reported information such as lifestyle habits is also collected by using same questionnaire items.

Materials and Method section (Page 5, Line 99)

The protocol of the Iwaki cohort study has been presented [4].

***Comment 5.*** *In figure 3 & 4, the differences between B and C were understood, but the results for A and C seem to be very similar. Careful explanations are needed about the results for A and C.*

**Author Response:** Thank you for the indication. We have changed the description about the difference between results as follows:

Results section (Page 21, Lines 229-235)

~~Both CHDs and stroke had a proportion of residents with low-risk scores that was higher in the CHAP scenario (Figs 3 and 4, respectively).~~ Under the hypothetical scenario without CHAP (Figs 3B and 4B, respectively), the proportions of those with low-risk scores for CHDs and stroke were gradually decreased since 2012 (Figs 3A and 4A, respectively), while they were remained at the same level under the actual scenario with CHAP (Figs 3C and 4C, respectively).

***Minor comment 1.*** *Page 16; line 196-197. This figure legend showed the definitions of risk groups in Figure 3. It should be in Figure 3.*

**Author Response:** Thank you for the indication. We have corrected as follows:

Fig 2 (Page 18, Lines 207-210)

Fig~~ure~~ 2. The Trend of Risk Scores. Error bars indicate 95% confidential intervals.

(A) CHDs and (B) Stroke.

~~Low1, ≤ 35; Low2, 36–40; Middle1, 41–45; Middle2, 46–50; Middle3, 51–55; High1, 56–60; High2, 61–65; High3, ≥ 66~~

Fig 3 (Page 22, Lines 236-239)

Fig 3. The Predicted Distribution of Risk Scores of CHDs (A) in 2012, (B) Predicted for 2021 in the Scenario Without CHAP, and (C) with CHAP.

Low1, ≤ 35; Low2, 36–40; Middle1, 41–45; Middle2, 46–50; Middle3, 51–55; High1, 56–60; High2, 61–65; High3, ≥ 66
