# Supplementary material for: Reduction in medical costs for cardiovascular diseases through innovative health awareness projects in a rural area in Japan
Source: PLoS One. 2022 Nov 16;17(11):e0277600. doi: 10.1371/journal.pone.0277600 (PMC9668126; doi:10.1371/journal.pone.0277600)
Supplement: Attachment — Submitted filename: response_to_reviewers_economic_impact_23Sep2022.docx [file pone.0277600.s004.docx]

Dear Reviewers,

Thank you for your careful reviews and constructive comments. We have revised the manuscript according to your suggestions and would be grateful if you would review the updated version.

Sincerely,

Ayako Shoji

**Reviewer #1**

***Comments to the Authors***

*Overall, most comments have been addressed, however there are still two points of major concern.*

***Comment 1.*** *It was previously suggested to use a before and after econometric method to explain the clinical and economic impact of the CHAP programme. The author responded saying that “Because CHAP was intended to change overall attitudes and behaviors of participants and it has been offered all participants since 2013, therefore we did not directly compare between the periods by using one model”.*

*The all point of a public health intervention such as the CHAP programme is to change overall attitudes and behaviours of participants. So, it is still not clear why a single before and after econometric model was not used. Also, it is understood that “the period with CHAP was set from 2012 to the end of the observation period” and that CHAP was offered to all participants since 2013. In this case, is not clear how the trend for risk score can be evaluated in the “before” model. If a single before -after econometric model cannot be used, I would have expected to see a risk score calculation at baseline (before CHAP introduction) then a “trend for risk score” for the years following the introduction of CHAP.*

*It maybe that this is provided in the existing analysis, but it is not clearly presented or framed.*

*On a minor note, the sentence could have been constructed better (e.g. “therefore” after “because” is not correct grammar).*

**Author Response:** Thank you for noting that our previous response did not adequately address your concerns, which we would like to rectify. As written in the previous response, we thought that it would be difficult to draw a comparison between the period prior to and the period following the initiation of CHAP by using a single model. CHAP aims to enhance individual participants’ dedication to a healthy life only once per year; we should consider the effects individually, but it was not possible due to a lack of patients who continuously participated throughout the whole program. However, we understand and appreciate the importance of your suggestion. Based on it, we created a single before and after model which considers the interaction effect between CHAP initiation and year. It indicated a significant effect of CHAP initiation on change in risk score trends. We have added the results as a new table in the manuscript (Table 2D) and provided explanatory descriptions as follows:

Materials and Methods section (Page 9, Lines 176-178)

To confirm the effect of the CHAP initiation on the trend of risk scores, we also used a single before and after model considering an interaction of the trend and the period.

Results section (Page 18, Lines 236-239)

A before and after model showed the decrease in risk scores of both CHDs and stroke associated with the interaction between years elapsed and the CHAP initiation, which suggested that the CHAP initiation contributed to significant decreases in risk scores of both CHDs and stroke (Table 2D).

Results section (Page 20, Line 244)

Table 2 (D)

We have also updated the whole manuscript according to a native English speaker’s advice.

***Comment 2.*** *This comment refers to an issue not previously picked up but of a major concern:*

*On page 7 (line 133-136 of the revised copy) the following is stated: “Participants who lacked checkup results of risk factors for CHDs and stroke were not excluded. We assumed no risk and counted a score of 0 for every missing risk factor. Detail information is shown in Supporting information”. It is not clear what this means. Are there missing records (e.g., if a patient did not have a risk factor for CHDs, 0 should be reported) or simply if the risk factors were not identified they were not reported? I believe that latter applies. In this case, I would simply delete the whole sentence, somewhat misleading.*

*However, if the former applies, assuming that patients with missing records on risk factors have 0 risk is quite a big assumption. This “dilutes” the proportion of patients with none or little risk. If it is a complete case (but this should be stated) the missing records should be reported and discarded. Alternatively, a multiple imputation should be carried out. It is appreciated that this for whatever reason may not be feasible, but if so, it should be clearly stated. At the minimum, the number of patients with missing records should be reported, and a sensitivity analysis excluding these patients should be conducted.*

**Author Response:** Thank you for your detailed feedback. We assumed that participants possessed zero risk for factors with missing records, which you correctly indicated as a possibility. We agree with your assessment that such an assumption can cause a serious bias in the estimates displayed in Table 2. Therefore, we conducted an additional sensitivity analysis in which we removed all participants with at least one missing record. This analysis confirmed the validity of our assumption by providing similar results to our original findings, as shown in Table S5. We furthermore amended the following sections, to include this information:

Materials and Methods section (Pages 7-8, Lines 139-141)

We confirmed the validity of this assumption via a sensitivity analysis in which we excluded participants with at least one missing data in the risk factors.

Results section (Page 17, Line 221)

NA shows the number of missing records in each risk factor.

Results section (Page 18, Lines 231-233)

A sensitivity analysis showed that results were similar even after removing all participants with at least one missing record (Table S5A and S5B).

Supporting information (Pages 5-6, Lines 27-33)

**Factors related to changes in risk scores after the removal of cases with missing records**

We removed participants with missing records of risk factors (the number of missing values of each factor is shown in Table 1) and examined the impact of our assumption that participants possess no risk in instances where records are missing for risk factors. All resultant estimates were similar, regardless of whether such patients were included or excluded in calculations (Table 2A and S5A and Table 2B and S5B).

Supporting information (Page 6, Lines 34-36)

Table S5. Factors related to risk scores.

**Reviewer #2**

***Comments to the Authors***

*All comments have been addressed. Authors replied adequately to my questions. I think the revise manuscript is much better.*

**Author Response:** Thank you for the confirmation that you are content with our revised manuscript.

**Reviewer #3**

***Comments to the Authors***

*After reviewing the revised version of this manuscript, I believe that authors have satisfactorily answered the queries raised by reviewers. The manuscript is well-written, has excellent-quality data and statistical analyses. I have only but one minor suggestion, that is:*

*In lines 36,37 of the abstract, authors state that "CHDs and stroke were significant even after CHAP (+ 0.413, p < .001; + 0.169, p < .001, respectively), but slightly more compared to before CHAP" I believe that authors meant to write "slightly less" instead of "slightly more".*

*I also recommend authors to perform a final check on English and grammar.*

**Author Response:** Thank you for reviewing our revised manuscript. As you indicated, the description was wrong and we have corrected it as indicated below. We have also updated the whole manuscript according to a native English speaker’s advice.

Abstract section (Page 2, Line 37)

After adjusting for sex, age, outside temperature on the checkup date, the preparation effect of CHAP in 2012, and risk scores in the previous year, the annual increases in risk scores of CHDs and stroke were significant even after CHAP (+0.413, p < 0.001; +0.169, p < 0.001, respectively), but slightly ~~more~~ less compared to before CHAP (+2.638, p < 0.001; +1.155, p < 0.001, respectively).
